# On the Influence of Maternal Shock in the Production of Fœtal Monstrosities

**Published:** 1880-10-20

**Authors:** Thomas Wilson


### ON THE INFLUENCE OF MATERNAL SHOCK IN THE PRODUCTION OF FŒTAL MONSTROSITIES.

BY THOMAS WILSON, M.R.C.S., L.R.C.P.

[Read before a conjoint meeting of the Border Counties and North of England branches of the British Medical Association.]

Most of us who are engaged in the routine of general practice, and especially in the department of midwifery, have had our attention repeatedly drawn to the occurrence of cases of abnormal development, for which the mother nearly always offers us an explanation. There are few of us, indeed, who have not ere this made acquaintance with such marks on children as nævi, the so-called "port wine stains," or "strawberry marks," and who have not received from the lips of the mother a theory of their causation.

As in the physical world every state of matter is conditioned by antecedent changes, or, in other words, is the effect of certain causes, so in the world of life there can be no such thing as a "freak of nature," no such thing as a spontaneous divergence from any line of development, without a concurrence of antecedent conditions, in which its cause may be said to lie.

It is out of a recognition of this fact that there has sprung up the theory of "maternal impressions," by which mothers and many others seek to explain the occurrence of abnormalities in their offspring by the operation of certain physical agencies. It was enough to the mother who had carried her child into the months of autumn, and who was then safely delivered of a baby with a large nævus on its face, to remember the fact of her visit to the market, and the effect which a large basket of strawberries produced on her imagination on that occasion. With many other similar remarks we are all more or less familiar, and how have we regarded them? Simply as crudities of thought, simply as the expression of human nature to find a cause for everything; simply as the wish, on the part of the maternal mind, to corroborate, in some way or other, the occurrence of certain peculiarities, in the skin of her child for instance, with certain objects in the external world presenting to her mind similarities of appearance. We all know how disinclined we have been to accept such statements, which would make the relationship that of cause and effect, knowing the impossibility for the imagination of the mother, by brooding over certain circumstances, to develop such changes in the child. Creation by fancy, so to speak, cannot occur—and hence we have come to consider certain conditions, which in the mother's mind stand related as cause and effect, as simple coincidences, unexplained, it is true, by any law with which we are familiar. We have preferred taking this view of the matter, on the testimony of our best anatomists, that "no nervous communication is known to exist between the mother and her fœtus in utero." Whatever effect external objects may have had on the imagination of a mother, it is evident that no amount of thought or reflection on the part of the maternal mind can develop abnormalities in the fœtus in her womb; for to assert the affirmative is simply to make the mind of the mother a creative faculty.

This is the position I have maintained, and yet the occurrence of certain cases in my practice has caused me to reconsider the question, and to arrive at this conclusion—that whether or not there be nervous communications between the mother and the child she is carrying in her womb, there is sufficient evidence pointing very strongly in the direction of the products of conception being affected by circumstances which produce shock to the mother; and without being too dogmatic on that point, it would appear that the earlier in tne period of utero-gestation, the greater is the liability to the product being affected. How it comes about that shock to the mother should thus interfere with the development of her child I cannot tell. I offer no theory, in the face of anatomical obstacles, but simply bring before you a few facts to show you that while no nervous communication is known to exist between mother and child, in utero, the effect of shock upon the mother is not confined to herself, but also acts upon the babe she is carrying. With this object in view, I shall briefly detail the facts of five cases.

*Case* 1.—Mrs. O——, aged twenty-five years, on giving birth to her second child, it was noticed that the child had spina bifida and club feet. The cause assigned by the mother was that when in the fifth month of pregnancy, she saw a man fall down in the street in an epileptic fit. The man in question was club footed.

*Case* 2.—Mrs. T——, aged twenty-three years, primapara; her child was born with a red mark on its back, and also a mark on the center of his forehead. Explanation given by the mother: when in her fourth month, a girl came behind her unawares and gave her a slap on the lumbar region; a month later, while reaching for some crockery in a closet, something fell down from above on her forehead.

*Case* 3.—Mrs. R——, aged thirty-four years, told me that in her sixth pregnancy, when about half gone, she was standing looking out of her doorway, and she saw a horse and cart run over a child's forehead; her own child's head was deficient from the eyes upwards.

*Case* 4.—Mrs. M——, aged sixteen years; her child was born with two distinct vesicles on the left hand, one over the metacarpal bone, and the other on the phalanx of the left index finger. Mother stated that a few days before the child was born she was ironing some clothes, while something startled her and she burnt her hand; the vesicles on the hand of the mother exactly corresponded with those on the hand of the child.

*Case* 5.—Mrs. W——, aged twenty-four years; her second child was born on the 28th of December, 1879, after a short labor of two-and-a-half hour's duration. It moaned from the time of its birth, and only lived two days. During this time it was unable to take the breast, and continued to moan until it died. There was, therefore, nothing unusual about the birth. As her first child only lived a fortnight, and this, her second, had died so soon after its birth from some unexplained cause, I suggested a post-mortem, with the following results. Autopsy: Body is that of a well-developed child. The face is pale, the fingers and finger nails are black, the fingers are not clinched. Rigor mortis well developed. Head: on reflecting the scalp it is noticed that the upper and posterior portions of the parietal and parieto-occipital regions are found to be of a very dark color. The colorarion is that which is noticed when blood has been effused into tissues, as in ecchymosis. The calvarium, on being removed, carried with it the dura mater, which adhered to the fontanelles. Under the arachnoid and over the left occipito-parietal lobes of the brain there is a good deal of blood effused. There are numerous small hemorrhages, varying in size from a pin's head to a pea; a similar condition exists under the membranes on the right side. The membranes, however, are easily removed, leaving the brain uninjured. The vessels are distended, on some there are dilatations. The membranes of the cerebellum are also hyperæmic. The brain and cerebellum healthy. The puncta hemorrhagica are not in any way increased. No effusion in ventricles, a little at the base of the brain. There was rupture of the lateral sinus. Chest : both lungs are engorged, especially in the lower lobes. They feel firm to the touch, and cut like a piece of flesh. The section is studded here and there with small reddish black points, as if of pulmonary apoplexy. The condition of the lung is that met wtth in the early stage of pneumonia. Heart : the left ventricle contains clotted blood; the right is also filled with clot, which can be traced into the pulmonary artery; the abdominal organs are healthy. There was no disease of blood-vessels. This was considered a case of cerebral pneumonia.

*Remarks.*—After some conversation with the mother the following explanation was given by her : In the act of removing something out of a large chest, some three months before her confinement, the lid came down suddenly on the crown of her head, and as it was somewhat heavy it caused her to faint, and to complain of pain in that region. Until a few days ago she complained of a dizziness in her head. Now, as nothing occurred during, or after, parturition to produce such a general ecchymosis under the scalp, and, so far as I am aware, there is no ante-partum condition which can cause it, I leave it to you to consider how much reliance is to be placed on the fact of injury to the mother.

We are in this condition with the five cases I have read to you. On the one hand we have certain abnormalities in some children, and, on the other, we have certain statements by their mothers. How do these two circumstances stand related to each other? Is it, as the mother insists, one of cause and effect? Is it possible that the mere observation of a person in a fit so impresses a woman that the child she is carying exhibits distinct signs of that at which she shuddered? Is the anencephalous monster, or the child with the spina bifida, but the material reflection of the maternal impression? (not after the manner of the mother brooding over the fact, and making herself miserable.) Is the visual and tactile sensation, which so impressed and sent a shudder through her at the time, capable of being followed by abnormal development in her child through the medium of the vaso-motor system?—*Obst. Journal of Great Britain and Ireland.*

